# An Evaluation of 1-Deoxynojirimycin Oral Administration in Eri Silkworm through Fat Body Metabolomics Based on ^**1**^H Nuclear Magnetic Resonance

**DOI:** 10.1155/2016/4676505

**Published:** 2016-05-11

**Authors:** Chao-wei Wen, Xiao-dong Lin, Min-jian Dong, Ming-jie Deng

**Affiliations:** ^1^School of Laboratory Medicine and Life Sciences, Wenzhou Medical University, Wenzhou 325035, China; ^2^Analytical and Testing Center of Wenzhou Medical University, Wenzhou 325035, China

## Abstract

1-Deoxynojirimycin (DNJ), the main hypoglycemic constituent in mulberry (*Morus alba*) latex, has been extensively researched. Although there is considerable interest in the biological effects of DNJ, the roles of 1-deoxynojirimycin (DNJ) in glycometabolism and energy metabolism in insects have received little attention. In this paper, ^1^H nuclear magnetic resonance (^1^H NMR) based metabonomic was performed to study the effects of the oral supplementation of 0.25% DNJ, 0.5% DNJ, latex, and the mixture of 0.5% DNJ and latex (1 : 1) on the fat body glycometabolism and energy metabolism of the fourth-instar larvae of Eri silkworms,* Samia cynthia ricini*. Metabolic pattern recognition analysis (partial least square-discriminant analysis, PLS-DA) of fat body extracts indicated that the groups of 0.25% DNJ, 0.5% DNJ, latex, and the mixture of 0.5% DNJ and latex (1 : 1) were significantly different from the control group. Further, compared to the control group, the metabolites levels of lactate, trehalose, succinate, malate, and fumarate were remarkably changed in experimental groups, which were involved in glycolysis, hydrolysis of trehalose, and tricarboxylic acid (TCA) cycle. Our results indicate that DNJ has a positive impact on the reverse energy metabolism of Eri silkworms and metabonomic analysis based on NMR can be used as a tool to identify potential biomarkers.

## 1. Introduction

Mulberry (*Morus alba*) trees have long been cultivated in China and the leaves have been used to rear the silkworm* Bombyx mori *[1]. Mulberry leaves exuding latex contained a number of traditional Chinese herbal medicines. One of the main active compounds in mulberry latex is the natural D-glucose analogue 1-deoxynojirimycin (DNJ) [2, 3]. DNJ has been reported to have an obvious effect to improve diabetic conditions by inhibiting the activity of *α*-glucosidase and the absorption of glucose in the intestinal brush border [4, 5]. The *α*-glucosidase inhibited competitively by DNJ leads to the impairment of some types of sugar metabolism and an increase in insulin sensitivity, preventing sugars from being hydrolyzed to *α*-D-glucose that humans can utilize [6, 7]. Based on this, DNJ inhibits postprandial intestinal glucose absorption and reduces the levels of blood sugar; thus it can be used for the treatment of diabetes. In recent years, increasing attention has been focused on the hypoglycemic effect of DNJ. However, the effect and regulatory mechanism of DNJ on glycometabolism of generalist herbivorous insects have been ignored.

Eri silkworms (*Samia cynthia ricini*, Saturniidae), with a short growth cycle, remarkable drug sensitivity, and well-studied food-related activities, provide a model for connections between pharmacological action and metabolism [8, 9]. Fat body is parallel to vertebrate adipose tissue and liver; it is not only the storage of nutrients but also the central organ of various substance metabolism in insects [10]. During the feeding stages, insects store glycogen and triglycerides as energy reserves in the fat body [11]. In response to energy demands, insects release diglycerides and trehalose from the fat body to hemolymph for utilization [12]. Overall, fat body has a close physical exchange with the hemolymph in vivo, which provides the energy for the life of Eri silkworms.

In our previous work, we had proved that metabolic profiles of hemolymph in Eri silkworms after oral administration of DNJ, latex, and the mixture of DNJ and latex (1 : 1) were significantly different compared with the age-matched controls. In addition, we also confirmed that only latex caused deaths of Eri silkworm, but not DNJ or the mixture [13]. However, further understanding of DNJ systematical regulation on energy metabolism in tissues of Eri silkworm has not yet been explored. In this study, we performed a NMR-based metabonomics analysis of the Eri silkworms fat body, after oral administration of DNJ or mulberry latex. Given the usefulness of NMR-based metabolomics technique in evaluating systemic responses to subtle metabolic perturbation, metabolomics can be used for the biological evaluation of DNJ as well as the identification of its potential medicinal properties. The results of this paper will be conducive to understand regulatory mechanism of DNJ on glycometabolism and energy metabolism.

## 2. Materials and Methods

### 2.1. Insects

In the experiment, Eri silkworms were maintained in the laboratory to identify the effects of mulberry latex and DNJ on non-mulberry-specialist caterpillars. Mulberry trees are not the natural host plant of* S. cynthia ricini* larvae.* S. cynthia ricini* larvae have been applied to detect and assess the levels of plant defense responses to herbivorous insects [1, 14]. In the experiment, we adopted newly molted fourth-instar larvae* S. cynthia ricini* (obtained from the Sericultural Research Institute of Chinese Academy of Agricultural Sciences, Zhenjiang, and then maintained in our laboratory of Wenzhou Medical University, Wenzhou) to evaluate the effects of mulberry latex and DNJ on this herbivorous insect. Hatched larvae were reared on castor oil leaves ad libitum until the end of the experiments underregulated temperature (20–25°C) and humidity.

### 2.2. Mulberry Latex

In this work, mulberry latex was gathered from the wild plants of* Morus alba* in the cultivated garden of Wenzhou Medical University, Wenzhou, China (28°N, 120°E) by directly cutting the petioles. The administration procedures of mulberry had been approved by the Institutional Plant Committee and Use Committee of Wenzhou Medical University (document number wydw2012-0083). The latex from this population contained only 0.32 ± 0% of DNJ and 1,4-dideoxy-1,4-imino-D-ribitol or D-AB1 was not detected in the latex [1]. The latex from the cut petioles was gathered with test tubes, maintained at 4°C, and then used in 12 h.

### 2.3. Experimental Design and Sample Collection

Firstly, 200 newly molted fourth-instar larvae were randomly selected, divided into four groups, and then fed with 0.25% DNJ (J&K Chemicals, Beijing, China), 0.5% DNJ, latex, and the mixture of 0.5% DNJ and latex (hybrid 1 : 1) at a single dosage of 5 *μ*L. The four groups were, respectively, named the 0.25% DNJ group, 0.5% DNJ group, latex group, and mixture group. The other 50 larvae were fed with 5 *μ*L of ultrapure water (Millipore, Massachusetts, USA) as the control group. After continuous administration for 2 d, fat body was collected by dissecting the individual from each group on Day 3 and the whole fat body from 5 individuals were brought together to form one sample. The collected fat body was snap-frozen in liquid nitrogen and stored at −80°C until use.

### 2.4. Preparation of Fat Body Samples and Acquisition of ^1^H NMR Spectra

The preparation of the fat body extracts was based on the previous reference [15]. The frozen tissue was weighed and ground using an electric homogenizer with ice-cold methanol (4 mL/g) and distilled water (0.85 mL/g) at 4°C and the mixture was vortexed. Chloroform (2 mL/g) and distilled water (2 mL/g) were added and mixed again. After keeping on ice for 15 min, the homogenate was centrifuged at 1,000 g for 15 min at 4°C. The supernatant was extracted and lyophilized for about 24 h. The metabolite mixture obtained was then weighed and dissolved in 0.6 mL of 99.5% D_2_O for NMR spectroscopy. All ^1^H NMR experiments were carried out on a Bruker AVANCE III 600 MHz NMR spectrometer (Bruker, Munich, Germany), with a spectral width of 12,000 Hz. The acquisition time was 2.65 s per scan, and an additional 10 s relaxation delay was used to ensure full relaxation. The number of scans was 256. The spectra were zero-filled to 64 K, and an exponential line-broadening function of 0.3 Hz was applied to the free induction decay prior to Fourier transformation. All spectra were corrected manually for phase and baseline and referenced to the chemical shift of the methyl peak of alanine (CH_3_, 1.48 ppm) using Topspin (v2.1 pl4, Bruker Biospin, Munich, Germany) [16].

### 2.5. Data Refinement and Multivariate Pattern Recognition Analysis

After the spectra were corrected, with the Topspin 2.1 software package, each spectrum was segmented into different chemical shift regions with the same width of 0.01 ppm, which was equivalent to the region of *δ* of 9.5~0.5, for multivariate pattern recognition analysis. In the analysis, the spectra region corresponding to residual peak from water resonance (5.8–4.6 ppm) was removed to zero. The data of remaining spectral segments were exported to Microsoft Excel. Before multivariate analysis, the peaks should be normalized to the sum of spectrum. Then, the concentrations of the metabolites were expressed as relative peak areas. The metabolite data derived from the control and treatment groups were imported into SIMCA-P 12.0 software (Umetrics, Umea, Sweden) to perform partial least square-discriminant analysis (PLS-DA). In order to differentiate the metabolic profiles obtained with fat body samples of the four groups, one of the most popular supervised PR methods, PLS-DA, was adopted to process the data obtained from the fat body samples. Based on PLS-DA, metabolites which could differentiate the control group from each treatment group were identified and integrated. From the integrated data, the relative intensity of each metabolite was then calculated [17]. Each point indicated an individual spectrum of a sample and could be differentiated from other points with the first two principal components, PC1 and PC2. Thus, the data could be displayed via plotting with the scores of PC1 and PC2. In the plots, each point indicated a single NMR spectral region segment and the metabolites related to differentiating the groups were exhibited by corresponding loading plots [18]. A coefficient of variation plots showed the differences in the metabolites among the groups, which allowed the interpretation because the loadings resembled NMR spectra. The loading plots and score plots can complement each other. The goodness of fit and model validity were tested and computed by the parameters of *R* ^2^ and *Q* ^2^, where *R* ^2^ represented the sum of the square of the entire *X* and *Q* ^2^ was the fraction of cross-validation-explained variation with the increase of the reliability [19].

### 2.6. Statistical Analysis

In order to obtain significant differences among metabolic changes, we analyzed the normalized integral values with SPSS 13.0 software (SPSS Inc., Chicago, USA). Each experimental group was compared with the control group, respectively. The independent samples* t*-test analysis was performed for comparison of means in selected signals between two groups. In the statistical analysis, independent samples* t*-test was used to analyze the acquired data. If the* p* value was calculated to be lower than 0.05, the difference was considered to be statistically significant. Data were expressed as mean ± standard deviations (SD).

## 3. Results and Discussion

### 3.1. ^1^H NMR Spectra Analysis of Fat Body

**Figure 1:**
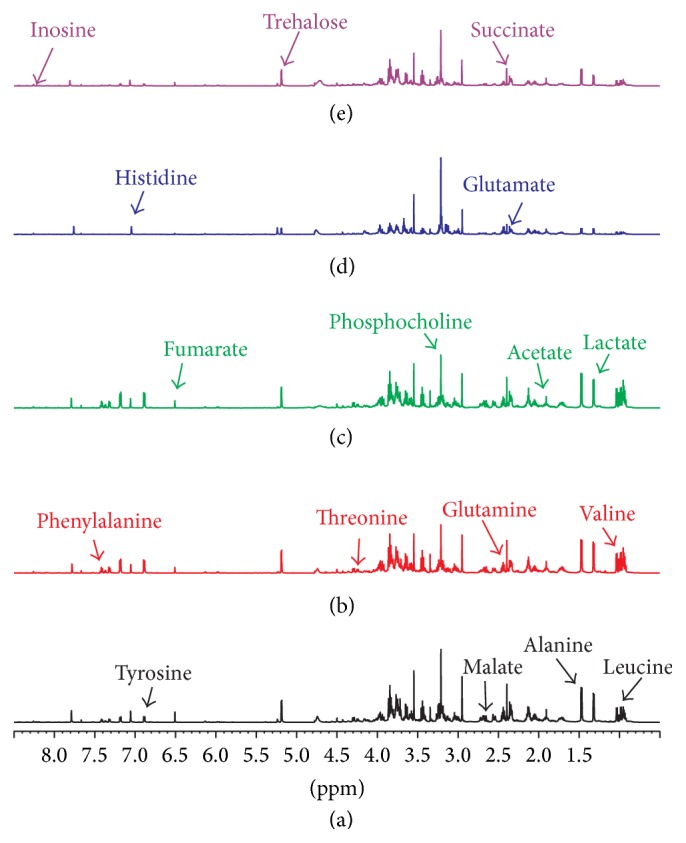
Representative ^1^H NMR spectra of fat body obtained from control Eri silkworms (a), 0.25% DNJ-fed Eri silkworms (b), 0.5% DNJ-fed Eri silkworms (c), latex-fed Eri silkworms (d), and mixture fed Eri silkworms (e).

Representative ^1^H-NMR spectra of fat body extracts obtained from Eri silkworms in the control and 0.25% DNJ, 0.5% DNJ, latex, and mixture of 0.5% DNJ and latex group are shown in Figures 1(a)–1(e). The spectral resonances of the metabolites were assigned according to our published work [18–21] and the 600 MHz library of the Chenomx NMR Suite 7.0 (Chenomx Inc., Edmonton, Canada). To confirm the assignments made from 1D ^1^H NMR spectra, some samples were also examined using 2D ^1^H–^1^H COSY spectra with solvent suppression. Endogenous metabolites, such as leucine (*δ*0.94), valine (*δ*1.04), lactate (*δ*1.33), alanine (*δ*1.47), acetate (*δ*1.91), glutamate (*δ*2.35), succinate (*δ*2.4), glutamine (*δ*2.45), malate (*δ*2.67), phosphocholine (*δ*3.22), threonine (*δ*4.25), trehalose (*δ*5.19), fumarate (*δ*6.51), tyrosine (*δ*6.88), histidine (*δ*7.04), phenylalanine (*δ*7.43), inosine (*δ*8.33), and trigonelline (*δ*9.12) were simultaneously measured through the ^1^H-NMR spectra of the fat body (Table 1).

### 3.2. Pattern Recognition Analysis of Fat Body

**Figure 2:**
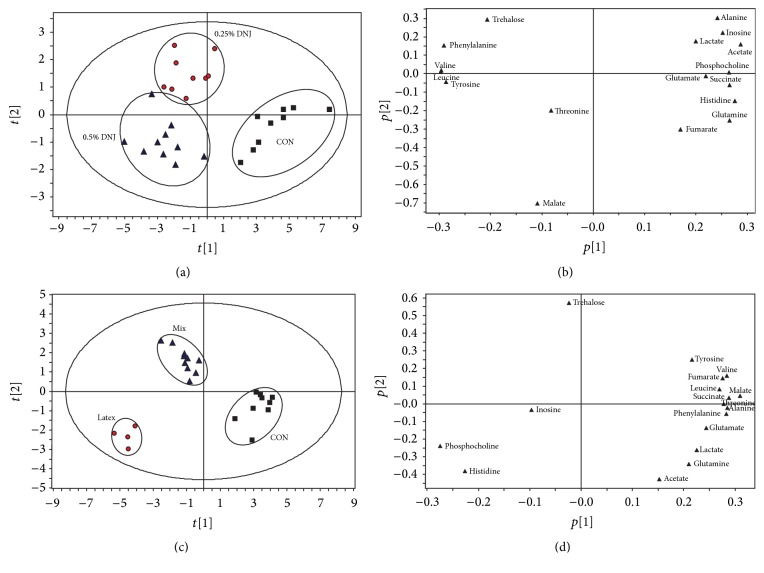
PLS-DA score plots (a) based on ^1^H NMR spectra of fat body from Eri silkworms of 0.25% DNJ group, 0.5% DNJ group, and control group (*R* ^2^ *X* = 0.714; *R* ^2^ *Y* = 0.774; *Q* ^2^ = 0.722; black square: control group, red circle: 0.25% DNJ group, and blue triangle: 0.5% DNJ group) and coefficient-coded loading plots (b). PLS-DA score plots (c) based on ^1^H NMR spectra of fat body from Eri silkworms of latex group, mixture group, and control group (*R* ^2^ *X* = 0.734; *R* ^2^ *Y* = 0.903; *Q* ^2^ = 0.882; black square: control group, blue triangle: mixture group, and red circle: latex group) and coefficient-coded loading plots (d).

To extract more details about DNJ-induced changes in the various metabolic systems and identify the potential metabolic pathways associated with the effect, fat body NMR spectra were segmented and subjected to partial least square-discriminant analysis (PLS-DA, Figure 2). As shown in Figure 2(a), clear discrimination along the PC1 direction was observed between 0.25% DNJ-fed Eri silkworms and 0.5% DNJ groups (*R* ^2^ *X* = 0.714, *R* ^2^ *Y* = 0.774, and *Q* ^2^ = 0.722); in the meanwhile, these two groups were almost separated from their age-matched controls along the PC2 direction, which shows that the cluster of 0.25% DNJ and 0.5% DNJ groups has diverse features compared to the control groups.  Figure 2(b) illustrates the corresponding loading plot with color-coded correlation coefficients (|*r*|) of metabolites between the DNJ groups and control groups and shows the variables responsible for the separation of different groups. The positive regions in the loading plot corresponded to metabolites that increased in quantity in the fat body of Eri silkworms, whereas negative regions amounted to metabolites that decreased in quantity in the fat body of Eri silkworms. Thus, the corresponding loading plot (Figure 2(b)) suggests that the separation was attributed to the variables, including acetate, valine, succinate, leucine, tyrosine, phenylalanine, phosphocholine, and histidine.

It is further demonstrated by the PLS-DA scores plot obtained from the fat body extracts of latex, mixture, and the control groups, where three groups are separated from each other along the PC1 direction or PC2 direction (Figure 2(c), *R* ^2^ *X* = 0.734, *R* ^2^ *Y* = 0.903, and *Q* ^2^ = 0.882). The clear separation of latex and mixture groups from the age-matched control suggests that metabolic perturbation occurs in the latex and mixture Eri silkworms. The corresponding loading plot (Figure 2(d)) shows that the variables that referred to phosphocholine, malate, alanine, threonine, valine, and histidine were the most responsible for the separation.

### 3.3. Changes in Metabolite and Metabolomics Study

**Figure 3:**
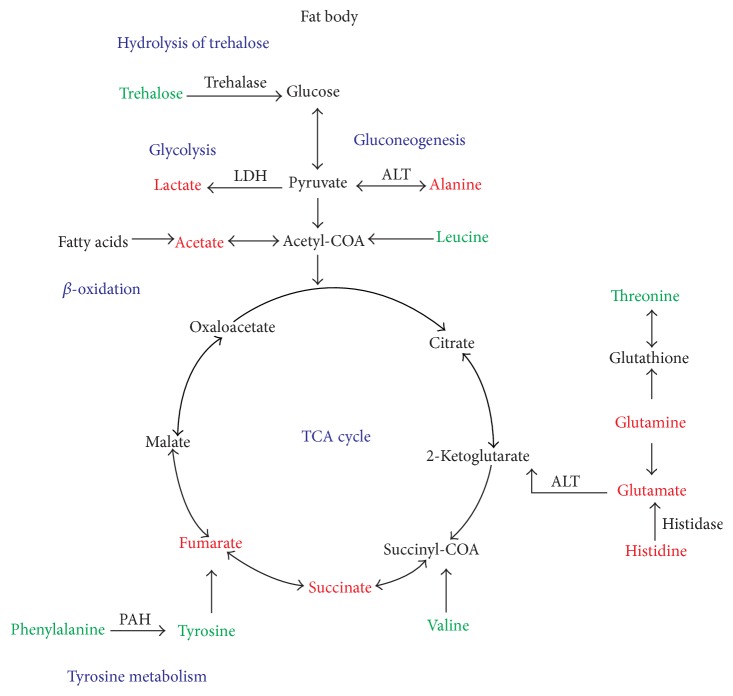
Schematic diagram of the metabolic pathways. The metabolite changes detected by ^1^H NMR fat body analysis and the pathway referenced to the KEGG database show the interrelationship of the identified metabolic pathways involved in the experimental Eri silkworms. Metabolites in red and green represent decrease and increase in DNJ (0.25% DNJ or 0.5% DNJ) groups, respectively, compared with control silkworms.

NMR is a powerful approach for studying tissue energy metabolism and glycometabolism and it has been previously used to investigate the changes in tissue extracts metabolism of various diseases, such as encephalopathies, nephropathy, and intestinal cancer [22–24]. In the present study, changes in the concentrations of fat body metabolites in Eri silkworms subjected to feed H_2_O, 0.25% DNJ, 0.5% DNJ, latex, and mixture by ex vivo ^1^H NMR spectroscopy were comprehensively reported. Obvious metabolic perturbations of biochemicals were observed, including some important products of glycometabolism and energy metabolism. DNJ-induced changes in the concentrations of metabolites exhibited notable features at 0.25% DNJ, 0.5% DNJ, latex, and mixture groups, respectively. Based on previous research, the metabolites altered and related pathways that appear to be regulated by DNJ may represent potential metabolic biomarkers for therapeutic targets and provide clues to elucidate the molecular mechanism of the agent acting on diabetes [5, 25–27]. In this paper, Table 1 shows the relative integral levels of metabolites in fat body samples from experimental and control Eri silkworms,* S. cynthia ricini*. The trend of the metabolic changes obtained by quantitative statistical analyses is in agreement with those indicated by the PLS-DA loading plots shown in Figure 2.  Figure 3 illustrates the altered metabolic pathways in fat body of Eri silkworms after oral administration of 0.25% DNJ and 0.5% DNJ based on the KEGG database (http://www.genome.jp/kegg/pathway.html).

Glycometabolism is essential for physiological balance of living organisms. In insects, trehalose is the major sugar and metabolic source of energy in the hemolymph and the hydrolysis of trehalose in two glycosidically linked glucose units is catalyzed by trehalase that is found in every insect tissue [28]. In the study, increased levels of trehalose related to the glycometabolism were observed in fat body of 0.25% DNJ, 0.5% DNJ, and mixture fed Eri silkworms. This finding suggested that the utilizable pathways of trehalose were inhibited by the potency of DNJ.

TCA cycle is the most effective way to get energy through carbohydrates or other substances oxidation in organisms. Its central importance to many biochemical pathways suggests that it is the hinge of carbohydrate, fat, and protein metabolism [29, 30]. In our work, decreased levels of succinate, malate, and fumarate related to TCA cycle were observed in fat body extracts of Eri silkworms in 0.25% DNJ, 0.5% DNJ, latex, and mixture groups. DNJ has been reported to be effective against hyperglycemia and lipid peroxidation in diabetics [31]. In the meanwhile, DNJ has a powerful effect on suppressing the virus and tumour activity, such as HIV, HBV, and melanoma cell [32–34]. Mulberry leaves exuding latex are highly toxic to* S. cynthia ricini*; latex plays key roles in defense against herbivorous insect except* Bombyx mori *[1]. The decrease in the relative intermediates concentrations of TCA cycle might be caused by the systemic stress generated under the physiological effects of DNJ and latex. It was known that these metabolites were intermediates of energy synthesis; as a result, decrease in the metabolites might induce less production of ATP [19]. In physiological conditions, tissues and cells obtain energy from the aerobic oxidation of sugar. Because aerobic metabolism of sugar is the most effective way to gain ATP in organisms, as its primary way, TCA cycle generates 24 ATP by one glucose molecule [35, 36]. Our results suggested that pathways of the TCA cycle and aerobic metabolism were impaired after oral administration of DNJ, latex, and mixture.

In addition, two additional glycolysis-related products, lactate and alanine, were observed decreasing in fat body after feeding 0.5% DNJ, latex, and mixture. As two of branched-chain amino acids (BCAAs), leucine and valine were enhanced in the 0.25% DNJ or 0.5% DNJ group but were reversed in the latex and mixture groups. Compared to the control, excretions of acetate, glutamate, phosphocholine, threonine, histidine, and inosine were markedly decreased in the DNJ groups, which were related to fatty acids *β*-oxidation, protein, lipid, purine, nucleic acid, and amino acid metabolism.

## 4. Conclusion

We demonstrated that metabonomic methods based on NMR could provide a useful tool for exploring biomarkers to elucidate pharmacological action discovered in biological chemistry. Our results indicated that DNJ impairs energy metabolism in body fat tissue of Eri silkworn. DNJ is a single-ingredient antidiabetic medicine due to bioactivity of regulating glycometabolism.

## Figures and Tables

**Table 1:** Metabolite normalized intensity of fat body obtained from newly molted forth-instar larvae control and age-matched experimental Eri silkworms,* S. cynthia ricini*.

*δ* ^1^H (ppm)	Metabolites	Metabolism pathway	H_2_O	0.25% DNJ	0.5% DNJ	Latex	Mixture
0.96	Leucine	BCAA metabolism	17.71 ± 3.34	26.31 ± 2.98^**∗∗**^	28.35 ± 3.57^**∗∗**^	10.11 ± 1.70^**∗∗**^	13.93 ± 2.06^**∗∗**^
1.04	Valine	BCAA metabolism	8.91 ± 1.82	13.01 ± 1.18^**∗∗**^	13.58 ± 1.29^**∗∗**^	4.01 ± 0.53^**∗∗**^	6.93 ± 0.59^**∗**^
1.32	Lactate	Glycolysis	8.09 ± 3.40	6.71 ± 1.18	5.40 ± 1.22^**∗**^	1.92 ± 0.87^**∗∗**^	2.25 ± 1.04^**∗∗**^
1.47	Alanine	Protein metabolism	26.01 ± 3.42	24.28 ± 2.24	20.93 ± 2.41^**∗∗**^	12.91 ± 1.37^**∗∗**^	19.59 ± 2.13^**∗∗**^
1.91	Acetate	Fatty acids *β*-oxidation	6.44 ± 0.44	5.77 ± 0.36^**∗∗**^	5.29 ± 0.30^**∗∗**^	6.13 ± 0.17	5.64 ± 0.47^**∗∗**^
2.35	Glutamate	Amino acid metabolism	33.36 ± 3.23	29.10 ± 1.27^**∗∗**^	27.46 ± 1.35^**∗∗**^	28.02 ± 0.73^**∗∗**^	28.41 ± 2.12^**∗∗**^
2.4	Succinate	TCA cycle	13.13 ± 1.61	9.64 ± 0.94^**∗∗**^	9.26 ± 0.98^**∗∗**^	5.49 ± 1.20^**∗∗**^	9.44 ± 1.53^**∗∗**^
2.45	Glutamine	Amino acid metabolism	26.15 ± 3.16	18.34 ± 1.09^**∗∗**^	19.68 ± 1.33^**∗∗**^	19.00 ± 5.05^**∗∗**^	16.48 ± 0.45^**∗∗**^
2.67	Malate	TCA cycle	10.39 ± 1.26	9.20 ± 0.58^**∗**^	12.09 ± 0.86^**∗∗**^	4.77 ± 0.77^**∗∗**^	7.24 ± 0.71^**∗∗**^
3.22	Phosphocholine	Lipid metabolism	33.25 ± 2.51	27.26 ± 2.82^**∗∗**^	25.55 ± 1.05^**∗∗**^	80.21 ± 3.73^**∗∗**^	44.67 ± 7.29^**∗∗**^
4.25	Threonine	Amino acid metabolism	6.15 ± 0.42	6.30 ± 0.35	6.54 ± 0.27^**∗**^	4.43 ± 0.30^**∗∗**^	5.27 ± 0.29^**∗∗**^
5.19	Trehalose	Glycometabolism	13.66 ± 1.91	17.05 ± 1.57^**∗∗**^	16.18 ± 1.66^**∗∗**^	10.05 ± 1.06^**∗∗**^	21.53 ± 2.29^**∗∗**^
6.51	Fumarate	TCA cycle	2.42 ± 0.38	1.97 ± 0.20^**∗∗**^	2.12 ± 0.31	0.98 ± 0.28^**∗∗**^	1.98 ± 0.26^**∗∗**^
6.88	Tyrosine	Amino acid metabolism	3.97 ± 1.15	8.36 ± 1.45^**∗∗**^	10.86 ± 2.17^**∗∗**^	1.58 ± 0.54^**∗∗**^	3.49 ± 0.94
7.06	Histidine	Purine metabolism	6.45 ± 0.34	5.34 ± 0.30^**∗∗**^	5.19 ± 0.37^**∗∗**^	9.79 ± 0.18^**∗∗**^	6.53 ± 0.49
7.43	Phenylalanine	Protein metabolism	2.96 ± 0.72	6.16 ± 0.76^**∗∗**^	6.03 ± 1.10^**∗∗**^	1.28 ± 0.16^**∗∗**^	1.59 ± 0.18^**∗∗**^
8.33	Inosine	Nucleic acid metabolism	0.24 ± 0.06	0.20 ± 0.03^**∗**^	0.15 ± 0.02^**∗∗**^	0.31 ± 0.13	0.30 ± 0.13

Values are expressed as mean ± SD.

^**∗**^ *p* < 0.05 and ^**∗∗**^ *p* < 0.01 indicate significant differences compared to the control group.
